# 50% Improvement: Should Treatment Response Go Beyond Symptom Improvement When Evaluating the Treatment of Depression?

**DOI:** 10.1192/j.eurpsy.2023.895

**Published:** 2023-07-19

**Authors:** M. Zimmerman

**Affiliations:** Brown University, Providence, United States

## Abstract

**Introduction:**

The emphasis on symptom resolution in depression treatment research is at variance with the recommendations of official treatment guidelines and the results of surveys of depressed patients’ views of the most important treatment goals.

**Objectives:**

In the present study, we examined the interrelationship between response rates on various outcome domains and whether response on each domain was associated with patients’ global rating of improvement (PGI) reported upon treatment completion. We also examined whether the PGI was associated with the number of domains on which the patients had achieved responder status and which domains were independent predictors of PGI response.

**Methods:**

Eight hundred and forty-four patients with major depressive disorder completed the Remission from Depression Questionnaire (RDQ), a self-report measure that assesses 6 constructs considered by patients to be relevant to assessing treatment outcome. The patients completed the RDQ at admission and discharge from the treatment program. For each domain, response was defined as a 50% or greater reduction in scores. At discharge, the patients rated the PGI.

**Results:**

The patients significantly improved from admission to discharge on each of the 6 domains assessed on the RDQ. The responders on each domain reported significantly greater improvement on the global rating of improvement at discharge. Responder status in one domain mostly co-occurred with responder status in another domain. In a logistic regression analysis, responses on all domains, except nondepressive symptoms, were independently associated with PGI response.

**Conclusions:**

The results of the present study are consistent with multiple surveys which have suggested that focusing on symptom reduction is too narrow of an approach when measuring outcome in the treatment of depression. Expanding the assessment of outcome beyond symptoms and viewing nonsymptomatic outcome domains as critical composites of primary endpoints would be more consistent with a patient-centered approach towards the treatment of depression.

**Disclosure of Interest:**

None Declared

